# Thirty-three-year follow-up of pseudoaneurysm of the mitral-aortic intervalvular fibrosa without surgical treatment: a case report and literature review

**DOI:** 10.1186/s13019-024-02885-7

**Published:** 2024-06-21

**Authors:** Seyed Mohsen Mirhosseini, Masood Soltanipur, Hossein Yarmohammadi, Mahdi Rezaei, Eisa Fattah, Fariba Bayat

**Affiliations:** 1 Cardiovascular Research Center, Shahid Beheshti University of Medical Sciences, Tehran, Iran; 2 Quality of Life Department, Breast Cancer Research Center, Motamed Cancer Institute, ACECR, Tehran, Iran; 3 Department of Mycobacteriology and Pulmonary Research, Pasteur Institute of Iran, Tehran, Iran; 4 School of Medicine, Shahid Beheshti University of Medical Sciences, Tehran, Iran

**Keywords:** Pseudoaneurysm of the mitral-aortic intervalvular fibrosa, PMAIF, Heart surgery, Nonsurgical treatment, Case report

## Abstract

**Background:**

Pseudoaneurysm of the mitral-aortic intervalvular fibrosa (PMAIF) is a rare complication of infective endocarditis or aortic valve surgery. Surgical treatment is suggested, but the long-term follow-up of conservative management remains unclear.

**Case presentation:**

A 33-year follow-up of a patient who developed PMAIF six years after aortic valve replacement is reported. The patient presented to our center with dyspnea, and the echocardiography revealed an ejection fraction of 20% and a PMAIF measuring 7 × 10 mm. Despite being advised to undergo surgery, the patient declined due to fear of surgical outcomes. Consequently, conservative treatment with close observation but without surgery was initiated. During the 33-year follow-up period, the patient did not experience any adverse health effects.

**Conclusion:**

Surgical intervention should be considered whenever the PMAIF is diagnosed. However, in any case that the surgery was not applicable, conservative management might lead to long-term survival, based on this and similar case reports in the literature.

**Supplementary Information:**

The online version contains supplementary material available at 10.1186/s13019-024-02885-7.

## Introduction

**Fig. 1:**
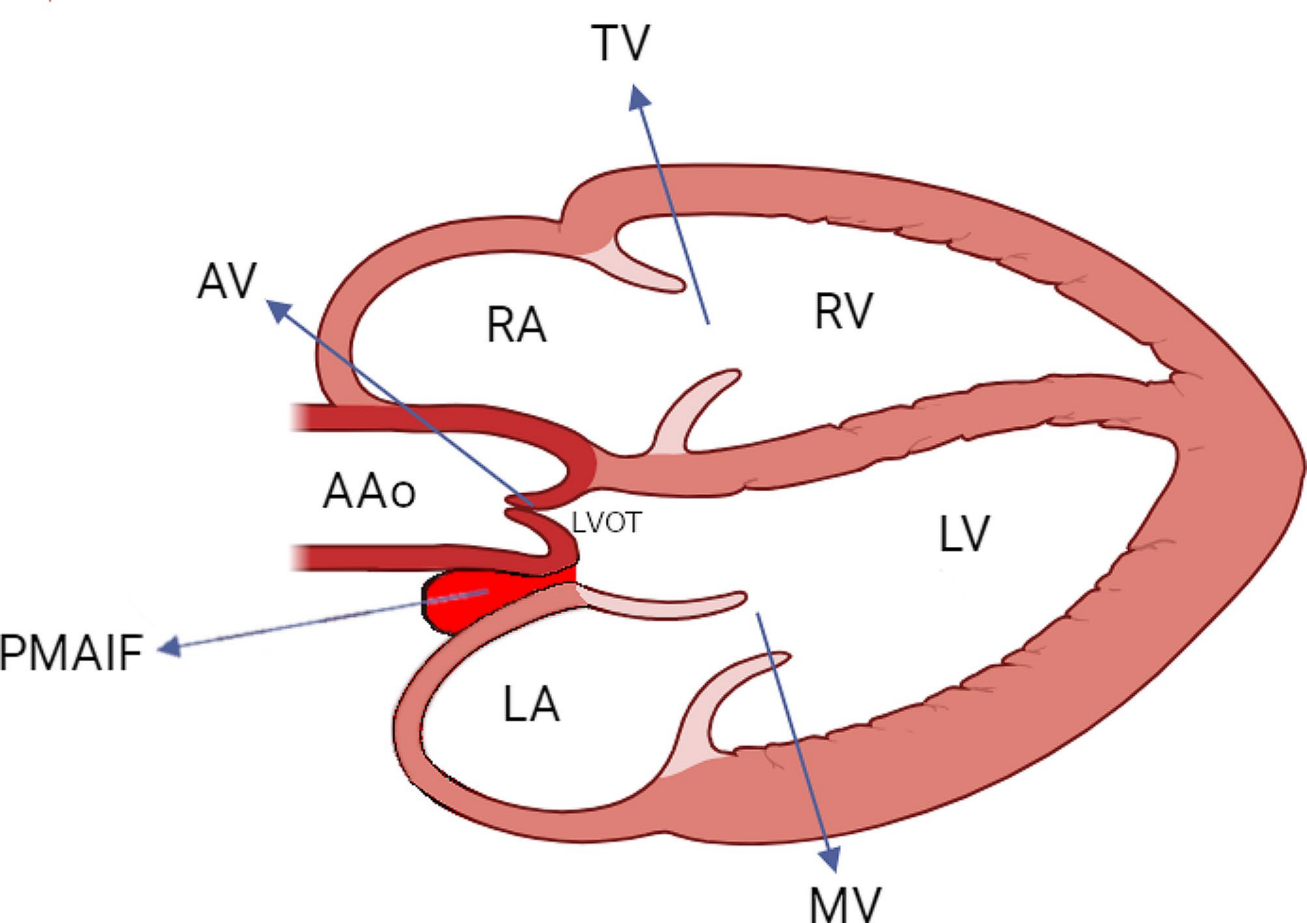
The schematic illustration of a PMAIF (arrow) **AAo**: ascending aorta, **LV**: left ventricle, **LA**: left atrium, **LVOT**: left ventricular outflow tract, **MV**: mitral valve, **PMAIF**: pseudoaneurysm of the mitral-aortic intervalvular fibrosa; **RV**: right ventricle, **RA**: right atrium, **TV**: tricuspid valve

The disease known as pseudoaneurysm of the mitral-aortic intervalvular fibrosa (PMAIF) is rare [1, 2]. This pseudoaneurysm is located at the aortic mitral curtain (AMC), which is a delicate and fibrous membrane that is situated between the aortic valve (AV) and the mitral valve (MV) [3]. This area serves as the connection point between the noncoronary cusp of the AV and the anterior mitral leaflet, and communicates with the left ventricular outflow tract (LVOT), as shown in Fig. 1 [2]. PMAIF is found in individuals with AV infective endocarditis (IE) or who have undergone aortic valve replacement (AVR) [4–6].

The prognostication of the clinical outcomes of PMAIF is challenging. It can be asymptomatic and uncomplicated or can be associated with complications such as rupture, embolization, and compression of essential anatomical components [7]. The occurrence of PMAIF rupture into the pericardium can be life-threatening [8]. Therefore, when PMAIF is identified, surgical intervention is advised for all patients, regardless of their lack of symptoms [7, 8]. The Commando or Combat technique is the standard surgical intervention for severe pathological processes such as IE that affect AMC structures (i.e. fistula, abscess, and pseudoaneurysm) [9]. Performing the Commando technique is challenging and complex. In this intervention, the objective is to replace the MV, the AV, and the AMC while reconstructing the LVOT using either the patient’s own pericardium (autologous) or donated tissue (allograft) [10]. When enough available tissue is lacking, anchoring the replacement prosthetic valve can present significant challenges [11]. Additionally, due to the extensive nature of the surgical intervention, both short-term and long-term death rates are significant [12]. In any case, which surgical intervention is not possible, a conservative therapeutic approach may be considered [7]. For these patients, close clinical observations and regular echocardiographic evaluations are necessary. Both transthoracic echocardiography (TTE) and transesophageal echocardiography (TEE) are recommended for follow-up evaluations [8, 13].

This article initially reports the long-term follow-up outcomes of a male patient with PMAIF following AVR who refused surgical intervention. Also, it aimed to gather evidence on the outcomes of PMAIF patients who did not receive surgery for this condition.

## Case presentation

The case describes a man who was born in the year 1960 and received a prosthetic metallic AV (bi-leaflet, 23 mm) due to severe aortic stenosis at the age of 25. He had no other significant past medical history except cranial surgery due to an extradural hematoma after a trauma in 1984, which did not damage his chest. He has been treated with warfarin 5 mg daily ever since, with an international normalized ratio (INR) of 2.5. Additionally, echocardiography was performed annually, and the ejection fraction (EF) was approximately 50% throughout those years of follow-up.

Six years after AVR, he started complaining of worsening dyspnea. He was referred to our center for the first time, and in the work-up, echocardiography revealed an EF of 20% and PMAIF with a size of 7 × 10 mm. During the consultation with cardiac surgery, he was informed that he needed to repair the pseudoaneurysm; however, he eventually refused to receive surgery since he was afraid of procedure outcomes. Therefore, conservative management with close observation was initiated. The medical treatment for heart failure included losartan 25 mg daily, carvedilol 6.25 mg bd, furosemide 40 mg daily, and spironolactone 25 mg daily. Blood profiles, including the INR, were checked every three to six months. He never had any episodes of IE, and serial blood culture at his first hospitalization in our center came back negative. Additionally, echocardiography was performed every six to 12 months. In some instances, TEE was performed to visualize PMAIF more accurately since TEE is better in determining structure posterior to the metallic AV.

**Fig. 2:**
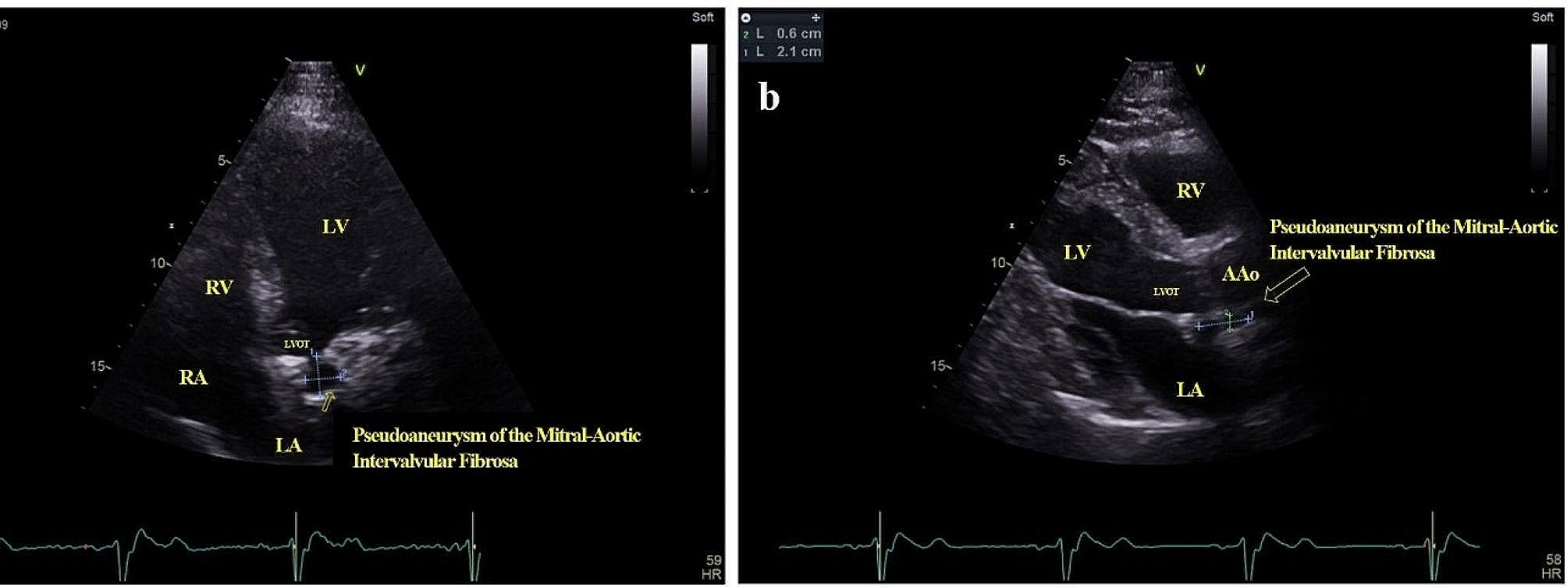
Transthoracic echocardiography revealed PMAIF: (a) four-chamber view of a PMAIF (arrow), size = 1.5 × 1.7 cm; (b) parasternal long-axis view of a PMAIF (arrow), size = 0.6 × 2.1 cm **LV**: left ventricle, **RV**: right ventricle, **RA**: right atrium, **LA**: left atrium, **LVOT**: left ventricular outflow tract, **AAo**: ascending aorta

**Fig. 3:**
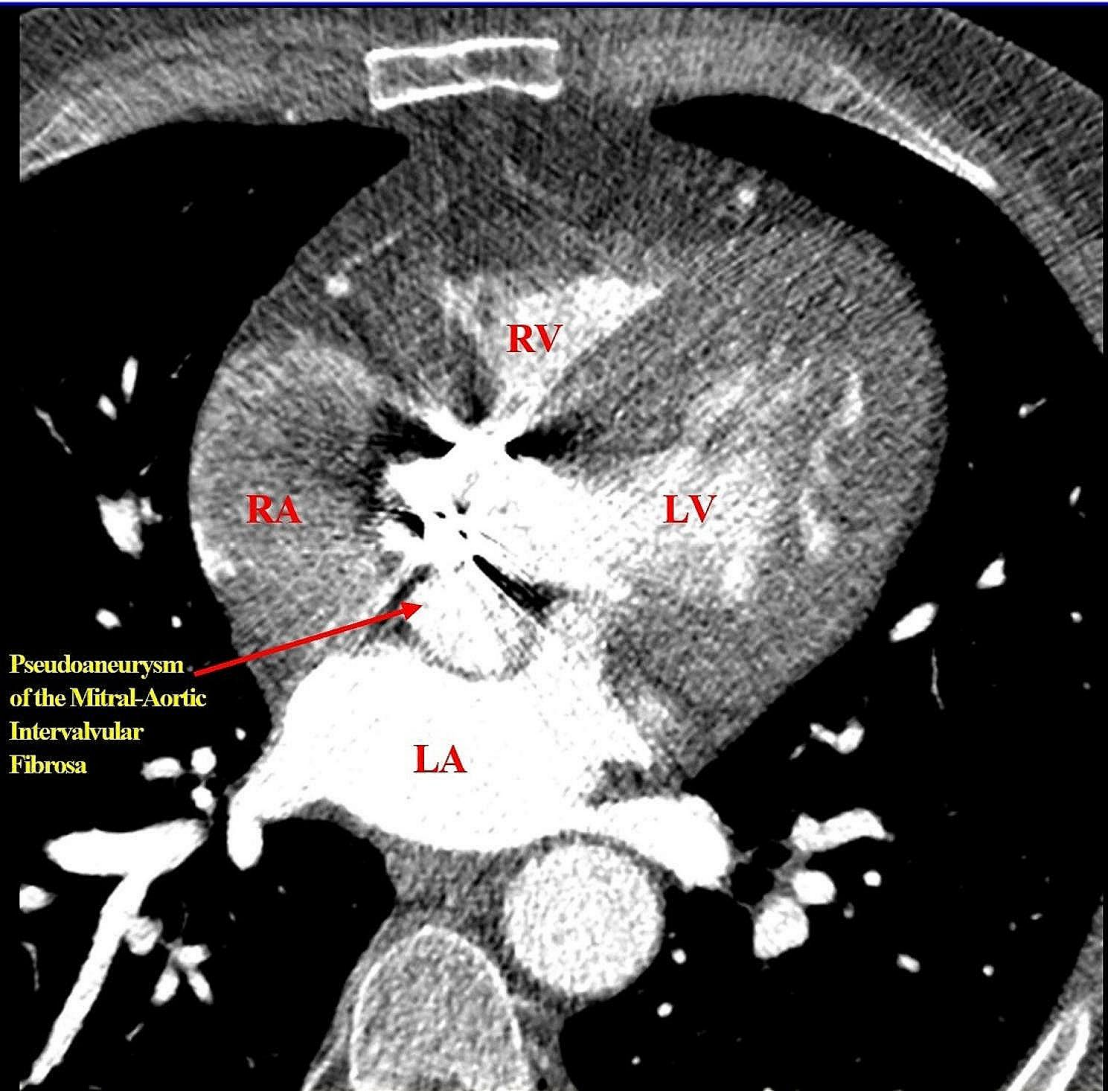
Contrast-enhanced computed tomography scan showing the PMAIF (arrow) in the axial plane, **LV**: left ventricle, **RV**: right ventricle, **RA**: right atrium, **LA**: left atrium

He has been followed up for 33 years, and interestingly, his prosthetic valve has remained functional. Regular follow-up echocardiography, which is presented in Table 1, shows a minimal increase in the size of PMAIF with a stable EF in the range of 25–30% during recent years. Figure 2 displays a TTE image of the patient. Also, the color Doppler echocardiography demonstrated the flow going to and out of the PMAIF during systole and diastole (the flow direction is marked by an arrow), as shown in Supplementary File 1. Furthermore, the contrast-enhanced computed tomography scan (CT scan) demonstrated the characteristic appearance of a PMAIF, as shown in Fig. 3.

**Table 1:** Echocardiography characteristics through 33 years of follow-up

Date	Echocardiography	LVEF	PMAIF Size	Function of mechanical aortic valve	Valvular disease	PAP
7/24/1991	TTE	20%	7 × 10 mm	N/A	N/A	N/A
10/05/2014	TEE	25–30%	Communication with LVOT	PPG: 44 mmHg MPG: 27 mmHg	Mild MR	25 mmHg
09/07/2015	TTE	25%	N/A	PPG:36 mmHg MPG: 17 mmHg	MR TR	N/A
09/15/2016	TTE	30–35%	N/A	N/A	MR	N/A
08/13/2017	TTE	35%	N/A	PPG: 45 mmHg MPG: 26 mmHg	MR	N/A
10/22/2018	TTE	20–25%	N/A	PPG: 40 mmHg MPG: 20 mmHg	Mild MR Mild TR	Normal
09/09/2019	3D TDI	20–25%	9 × 13 mm No leakage	N/A	Mild MR	N/A
11/27/2020	3D TEE + TTE	30–35%	11.3 × 15.9 mm Mild leakage	N/A	N/A	N/A
10/16/2021	TDI	25–30%	13 × 18 mm No connection	PPG: 36 mmHg MPG: 22 mmHg	Mild MR	Normal
10/14/2022	TDI	30%	16 × 20 mm Connection to LVOT (diameter 3 mm)	PPG: 32 mmHg MPG: 19 mmHg	Mild MR Mild to moderate TR	25 mmHg
10/22/2023	TDI	30%	15 × 17 mm Orifice 4.5 mm	PPG: 30 mmHg MPG: 17 mmHg	Mild MR Mild TR	30 mmHg

**TEE**: Transesophageal echocardiography, **TTE**: Transthoracic echocardiogram, **mm**: millimeter, **N/A**: Not available, **PAP**: pulmonary artery pressure, **MR**: Mitral regurgitation, **TR**: Tricuspid regurgitation, **PPG**: peak pressure gradient, **MPG**: mean pressure gradient, **LVOT**: Left ventricular outflow tract, **LVEF**: Left ventricular ejection fraction, **TDI**: Tissue Doppler imaging

The latest laboratory results were within normal or expected limits as follows: white blood cells: 5700 cells/mm^3^, red blood cells: 5,010,000 cells/mm^3^, platelet: 230,000 cells/mm^3^, hemoglobin: 14.0 g/dl, hematocrit: 40.5%, MCV: 86.8 fl., MCH: 29.9 pg, MCHC: 34.5 gr/dl, RDW: 13.3%, neutrophils: 73%, lymphocytes: 20%, monocyte: 5%, eosinophil: 1%, PT: 21.9 s, INR: 2.5, PTT: 45.2 s, fasting blood sugar: 94 mg/dl, blood urea nitrogen: 14 mg/dl, creatinine: 1.4 mg/dl. During these past years, he never had any history of cardiovascular disorders, cerebrovascular accidents, and, severe infection.

## Discussion and literature review

PMAIF is associated with both IE of the AV and artificial AV implantation [14]. IE which affects the AV is most commonly associated with the development of PMAIF [15]. Infections of the AV can potentially spread directly to the area of the AMC. The relatively avascular characteristic of AMC makes it susceptible to infection, leading to the formation of PMAIF [8]. The microorganisms *Streptococcus spp* and *Staphylococcus spp* are the predominant causative agents in such cases [15]. On the other hand, one of the most common traumatic surgeries is AVR which makes it the second leading cause of PMAIF formation [15]. Due to the close association of the AMC with the anatomy of the aortic root, it is at risk of damage during these procedures. Therefore, any abnormality in the AV observed in the echocardiography of a patient with a history of previous or recent AVR should prompt further investigation for a possible PMAIF [8].

While larger instances of PMAIF might be identified through TTE [8], the diagnosis of PMAIF typically necessitates TEE [16]. There have been recent advancements in diagnostic methods for PMAIF, such as three-dimensional echocardiography, cardiac CT scans, and magnetic resonance imaging (MRI). However, it remains unclear what additional value these techniques offer compared to standard two-dimensional TEE [17]. In our case, both TTE and TEE were used for PMAIF follow-up. The change in the size of PMAIF is an important parameter at follow-up, however, it is not clear whether the pseudoaneurysm increases in size before rupturing. Therefore, the possibility of a rupture and fatal consequences needs to always be considered. Other prognostic parameters evident on echocardiography are the paravalvular leak and PMAIF connections or fistula to LVOT and aorta [8, 15]. Other possible complications include rupture into the left atrium or aorta, localized compression of a coronary artery causing myocardial ischemia, distortion of the mitral valve leading to mitral regurgitation, and the development of blood clots and subsequent distal embolization [18].

Surgical intervention must be advised for PMAIF management to all symptomatic or non-symptomatic patients, yet the optimal survival outcomes remain to be achieved since it is a complex procedure and most of the evidence has been reported by professional tertiary cardiovascular surgery centers [12, 19]. Commando and Hemi-Commando procedures are the surgical interventions that are indicated for PMAIF treatment. The initial description of this procedure dates back to the year 1976 [20]. The methodology involves the replacement of the MV and AV, as well as the reconstruction of the LVOT using either autologous or allograft pericardium [10]. Also, the Hemi-Commando technique is an alternative approach used when the pathological process does not involve the entire MV. Unlike the Commando procedure, which involves repairing both the anterior and posterior leaflets of the MV, the Hemi-Commando procedure leaves the posterior leaflet of the MV intact [21, 22]. The repair procedure involves complex details. Initially, a diagonal incision towards the non-coronary sinus’s base is performed for aortotomy. Then a left atriotomy is performed which connects the right superior pulmonary vein to the anterior mitral leaflet. Following this, the AV is removed along with the excision of the anterior mitral leaflet and the AMC. The left ventricle is exposed in a triangular shape, allowing for the removal of the posterior mitral leaflet. Then sutures for the mitral prosthesis are passed through the posterior annulus. In the next stage, the AMC is reconstructed utilizing either the “double patch technique” or the “single patch technique”. On the other hand, in the Hemi-commando technique, a homo/allograft using to replace the aortic root, the AV, and the anterior leaflet of MV. The homo/allograft aortic conduit with anterior mitral leaflet is positioned as a unit, and finally, the MV ring annuloplasty is done [23]. However, this invasive procedure is associated with numerous complications. A recent meta-analysis, involving 540 patients with a median follow-up of about 41 months, revealed that this surgical intervention has significant early mortality (16.2%) and postoperative complications such as pacemaker implantation (25.1%), bleeding (13.1%), and stroke (7.8%). The long-term survival rate for these patients was reported at 57 ± 5% [12]. The outcome of this technique is influenced by several factors, such as the patient’s age, post-medical history, previous cardiac surgery, and the experience of the surgeon [19, 24, 25]. So, this type of surgical procedure should be conducted at specialized centers with a high level of expertise in the surgical technique [12].

Although the standard management of PMAIF requires surgical intervention, in some instances, it might not be possible, as in our case which the patient did not give consent for the procedure. Therefore, at this point the conservative management of PMAIF without surgical intervention is the choice, however, limited evidence exists on this. There are instances in earlier studies where patients with PMAIF refused surgery and remained asymptomatic and in good health throughout the follow-up period. These patients generally have favorable outcomes without experiencing adverse effects [26]. To gather the relevant evidence, a literature review was conducted. PubMed, Scopus, and, Web of Science were searched based on the relevant keywords. Any study reporting conservative management without surgical intervention was eligible to be included. Also, there was no limitation on the age, sex, and, etiology of the PMAIF. Case reports, case series, and, other retrospective or prospective studies were included, however, articles in other languages than English were excluded. Articles that had not provided information regarding the follow-up of the PMAIF patient were not excluded. To align our findings with evidence-based medicine and describe the trustworthiness of search results, we used the Joanna Briggs Institute (JBI) checklist for case reports [27]. Additionally, we determined the level of evidence for each included article based on the Oxford level of evidence (LoE) [28].

The literature review included 13 articles that reported nonsurgical methods for managing PMAIF, and the results are shown in Table 2. The results of the quality assessment based on the JBI checklist for case reports and Oxford LoE are presented in supplementary file 3 (supplementary Table 1). These studies included a total of 25 patients from 2008 to 2022 and can be found in supplementary file 2. A significant portion of the patients were male, accounting for 17 out of the 25 patients included. The age range of patients was broad, from 1 month to 87 years, indicating that diverse age groups need cardiac diagnostics and interventions. Among all the patients, 9 men had a history of surgery. These surgeries include AVR, mechanical valve implantation, coronary artery bypass grafting, aortic valvotomy, resection for subaortic stenosis, and the Bentall procedure. In addition, 5 surgical operations involving ventricular septal defect repair, AVR, and composite graft replacement of the ascending aorta have been reported for female patients. IE was absent in most patients. However, three patients had past histories of IE before their latest evaluations: two males aged 55 and 77 years old and one female aged 87 years old [7, 8, 15, 29–38].

**Table 2:** Demographics, clinical characteristics, and follow-up data of patients with PMAIF in published articles

First author (year)	Characteristics (Number of Cases/Age/Sex)	Prior surgery/IE	Symptoms/Diagnosis	Size (mm)	Follow-up (year/size changes)
Bishara et al. (2022) [29]	One / 16 y / male	None / None	None / TTE and TEE	N/A	3 years / Minor Changes
Niwano et al. (2021) [35]	One / 76 y / female	AVR / None	None / TTE, TEE, MRI, and CT-Scan	N/A	30 years / Minor Changes
Del Pasqua et al., (2019) [31]	One / 1 month / female	None / None	Innocent murmur / TTE, TEE, and CT-Scan	7 × 6 mm	5 years / 12 × 10 mm
Low et al. (2018) [34]	Five / ranged from 18 to 64 y / 5 males	Mechanical valve: 1 case / None	Arrhythmia: 2 cases / TEE: 4 cases & TTE, MRI, and CT-Scan: 5 cases	Ranged from 16 to 52 mm	4 years / Minor Changes
Caro-Dominguez et al. (2017) [30]	One / 13 y / male	None / None	Chest Pain / TTE and MRI	N/A	6 months / Minor Changes
Apostolidou et al. (2017) [15]	One / 84 y / male	CABG / None	Dyspnea / TEE	N/A	15 months / N/A
Han et al., (2016) [33]	One / 27 y / male	None / None	None / TTE	8 × 12 mm	3 years / Minor Changes
Bonou et al. (2015) [38]	Two / 42 y and 77 y / 1 male and 1 female	AVR: 2 cases, CABG: 1 case / IE: 1 case	Dyspnea and palpitations: 1 case & fever: 1 case / TTE: 1 case & TEE and CT: 2 cases	33 mm 22 mm	3–4 years / Minor Changes
Şahan et al. (2015) [8]	Three / ranged from 23 to 71 y / 1 male and 2 female	None / None	None / TTE and TEE: 3 cases	21 × 11 mm 12 × 31 mm 27 × 21 mm	3 years / 49 × 78 mm in the second case & minor changes in others
Hasin et al. (2011) [7]	Two / 43 y and 55 y / 2 male	AVR: 1 case, Aortic valvotomy: 1 case / IE: 1 case	None / TTE: 1 case & TEE: 2 Cases	40 mm 60 mm	5–16 years / 48 mm and 68 mm in order
Gin et al. (2011) [32]	Three / ranged from 31 to 87 y / 1 male and 2 female	AVR: 1 case, VSD Repair and Resection SAoS: 1 case / IE: 1 case	None / TEE: All cases	53 × 23 mm 76 × 49 mm 48 × 25 mm	3–9 years / minor Changes
Grimaldi et al. (2011) [37]	Three / Age range: 50 to 75 y / 2 male and 1 female	AVR and Bentall procedure for severe AI: 1 case, AVR + CGR: 1 case / None	None / TTE and TEE: 3 cases & CT-Scan: 1 case	15 × 20 mm 15 × 20 mm 40 × 50 mm	4 years / Minor Changes
Salerno et al. (2008) [36]	One / 82 y / male	AVR / None	Fever / TTE	62 mm	1 year / minor Changes

**AVR**: Aortic valve replacement, **AI**: Aortic insufficiency, **CABG**: Coronary artery bypass graft, **CT-Scan**: Compound tomography scan, **CGR**: Composite graft replacement, **IE**: Infective endocarditis, **MRI**: Magnetic resonance imaging, **mm**: Millimeter, **N/A**: Not available, **SAoS**: Subaortic stenosis, TEE: Transesophageal echocardiography, **TTE**: Transthoracic echocardiogram, **VSD**: Ventricular septal defect, **y**: years

Imaging and diagnostic tools included TTE for 19 patients, TEE for 21 patients, and MRI and CT scans for 11 patients. These tools were frequently used and mostly showed minor changes in aortic size over the follow-up periods, ranging from 6 months to 30 years. Regarding past medical histories, there was one patient with Bechet’s disease, one patient with Takayasu disease, and one patient with rheumatic heart disease. Furthermore, patients presented with a range of symptoms, including arrhythmias, fever, chest pain, and innocent murmurs. Ten patients presented with moderate and severe aortic regurgitation (AR), while moderate and severe mitral regurgitation (MR) occurred in four patients. Overall, four patients showed signs of both MR and AR [7, 8, 15, 29–38].

Based on the literature review, there have been other reports similar to our case, describing a conservative follow-up of PMAIF without surgical intervention. However, it should be remembered that case studies are among scientific reports with the lowest level of evidence in evidence-based medicine [28]. Therefore, despite the uneventful long-term follow-up in our case and other similar cases, surgical intervention must be advised to all patients with PMAIF. Our study was limited by incomplete and/or missing patient records. Additionally, our review was restricted to the English literature related to this rare disease entity. Nevertheless, this article presents our experience of conservative management for PMAIF without surgical intervention.

## Conclusion

Our case report described the long-term follow-up of a PMAIF patient who refused surgical intervention. The literature search also resulted from limited evidence regarding conservative management of PMAIF which most of these cases had no complications during the follow-up. Nevertheless, surgical intervention must be advised to all PMAIF patients despite being symptomatic or not. Future studies need to be focused on improving outcomes of the surgical intervention for PMAIF treatment.

### Electronic supplementary material

Below is the link to the electronic supplementary material.

Supplementary Material 1. mp4 Color Doppler view of the PMAIF (arrow).

Supplementary Material 2. xls Characteristics of 25 patients with PMAIF who underwent conservative management without surgical intervention in published articles.

Supplementary Material 3. pdf CARE checklist, quality assessment, and Oxford level of evidence.

## Data Availability

Data are available based on a request from the corresponding author.

## Declarations

### Standards of Reporting

A CARE checklist is provided in supplementary file 3 (supplementary Table 2) for reporting case reports.

## Abbreviations

AMC: Aortic mitral curtain
AR: Aortic Regurgitation
AV: Aortic valve
AVR: Aortic Valve Replacement
CT: scan Computed tomography scan
EF: Ejection Fraction
INR: International normalized ratio
IE: Infective endocarditis
JBI: Joanna Briggs Institute
LVOT: Left ventricular outflow tract
LoE: level of evidence
MR: Mitral Regurgitation
MRI: Magnetic resonance imaging
MV: Mitral valve
mg: milligram
mm: millimeter
PMAIF: Pseudoaneurysm of the Mitral-Aortic Intervalvular Fibrosa
TTE: Transthoracic Echocardiography
TEE: Transesophageal Echocardiography
