# Supplementary material for: Thirty-three-year follow-up of pseudoaneurysm of the mitral-aortic intervalvular fibrosa without surgical treatment: a case report and literature review
Source: J Cardiothorac Surg. 2024 Jun 21;19:345. doi: 10.1186/s13019-024-02885-7 (PMC11191241; doi:10.1186/s13019-024-02885-7)
Supplement: Supplementary file 3 — Supplementary Material 3. pdf CARE checklist, quality assessment, and Oxford level of evidence. [file 13019_2024_2885_MOESM3_ESM.pdf]

Supplementary table 1. Results of Critical Appraisal Using JBI Case-Reports Studies Checklist

Study	Q1	Q2	Q3	Q4	Q5	Q6	Q7	Q8	Total (%) Yes	Oxford LoE
Bishara *et al.*	Y	Y	Y	Y	Y	Y	Y	Y	**100%**	**4**
Niwano *et al.*	Y	Y	Y	Y	N	Y	Y	N	**87%**	**4**
Del Pasqua *et al.*	Y	Y	Y	Y	U	Y	Y	N	**75%**	**4**
Low *et al.*	Y	N	N	Y	Y	N	Y	Y	**62.5%**	**4**
Caro-Dominguez *et al.*	Y	Y	Y	Y	N	Y	Y	N	**75%**	**4**
Apostolidou *et al.*	Y	Y	Y	Y	Y	Y	Y	Y	**100%**	**4**
Han *et al.*	Y	N	N	Y	Y	U	Y	Y	**62.5%**	**4**
Bonou *et al.*	Y	Y	Y	Y	Y	Y	Y	Y	**100%**	**4**
Şahan *et al.*	Y	N	Y	Y	N	Y	Y	Y	**75%**	**4**
Hasin *et al.*	Y	Y	Y	Y	Y	Y	Y	Y	**100%**	**4**
Gin *et al.*	Y	Y	Y	Y	Y	Y	Y	U	**87%**	**4**
Grimaldi *et al.*	Y	Y	Y	Y	Y	Y	Y	Y	**100%**	**4**
Salerno *et al.*	Y	Y	Y	Y	Y	Y	Y	Y	**100%**	**4**
**Total (%) Yes**	**100%**	**76%**	**84%**	**100%**	**69%**	**84%**	**100%**	**69%**		

JBI: Joanna Briggs Institute, **N:** No, **NA:** Not Applicable, **U:** Unclear, **Y:** Yes, **LoE:** level of evidence.

Critical appraisal questions:

Q1. Were patient’s demographic characteristics clearly described?

Q2. Was the patient’s history clearly described and presented as a timeline?

Q3. Was the current clinical condition of the patient on presentation clearly described?

Q4. Were diagnostic tests or assessment methods and the results clearly described?

Q5. Was the intervention(s) or treatment procedure(s) clearly described?

Q6. Was the post-intervention clinical condition clearly described?

Q7. Were adverse events (harms) or unanticipated events identified and described?

Q8. Does the case report provide takeaway lessons?

Supplementary table 2. Results of Critical Appraisal Using CARE Case-Report Studies Checklist

Topic	Item	Checklist item description	Reported on Line
Title	1	The diagnosis or intervention of primary focus followed by the words “case report”	Yes
Key Words	2	2 to 5 key words that identify diagnoses or interventions in this case report, including "case report"	Yes
Abstract (No references)	3a	Introduction: What is unique about this case and what does it add to the scientific literature?	Yes
	3b	Main symptoms and/or important clinical findings	Yes
	3c	The main diagnoses, therapeutic interventions, and outcomes	Yes
	3d	Conclusion—What is the main “take-away” lesson(s) from this case?	Yes
Introduction	4	One or two paragraphs summarizing why this case is unique (may include references)	Yes
Patient Information	5a	De-identified patient specific information	Yes
	5b	Primary concerns and symptoms of the patient	Yes
	5c	Medical, family, and psycho-social history including relevant genetic information	Yes
	5d	Relevant past interventions with outcomes	Yes
Clinical Findings	6	Describe significant physical examination (PE) and important clinical findings	Yes
Timeline	7	Historical and current information from this episode of care organized as a timeline	Yes
Diagnostic Assessment	8a	Diagnostic testing (such as PE, laboratory testing, imaging, surveys)	Yes
	8b	Diagnostic challenges (such as access to testing, financial, or cultural)	No
	8c	Diagnosis (including other diagnoses considered)	Yes
	8d	Prognosis (such as staging in oncology) where applicable	Yes
Therapeutic Intervention	9a	Types of therapeutic intervention (such as pharmacologic, surgical, preventive, self-care)	Yes
	9b	Administration of therapeutic intervention (such as dosage, strength, duration)	Yes
	9c	Changes in therapeutic intervention (with rationale)	Yes
Follow-up and Outcomes	10a	Clinician and patient-assessed outcomes (if available)	Yes
	10b	Important follow-up diagnostic and other test results	Yes
	10c	Intervention adherence and tolerability (How was this assessed?)	Yes
	10d	Adverse and unanticipated events	Yes
Discussion	11a	A scientific discussion of the strengths AND limitations associated with this case report	Yes
	11b	Discussion of the relevant medical literature with references	Yes
	11c	The scientific rationale for any conclusions (including assessment of possible causes)	Yes
	11d	The primary “take-away” lessons of this case report (without references) in a one paragraph conclusion	Yes
Patient Perspective	12	The patient should share their perspective in one to two paragraphs on the treatment(s) they received	Yes
Informed Consent	13	Did the patient give informed consent? Please provide if requested	Yes
